# Dynamic Changes in Serum Aquaporin-1 and Aquaporin-5 in Transient Tachypnea of the Newborn

**DOI:** 10.3390/ijms27167243

**Published:** 2026-08-14

**Authors:** Abdurrahman Avar Ozdemir, Murat Ekinci, Gulsen Acar, Halime Sema Can, Gokhan Buyukkale, Ismail Dag

**Affiliations:** 1 Division of Neonatology, Department of Pediatrics, Kanuni Sultan Suleyman Training and Research Hospital, Istanbul 34303, Turkey; murekus@hotmail.com (M.E.); drgulsenacar@gmail.com (G.A.); semacanbuker@gmail.com (H.S.C.); gbuyukkale@hotmail.com (G.B.); 2 Department of Biochemistry, Eyüpsultan Hospital, Istanbul 34050, Turkey; isdag78@gmail.com

**Keywords:** transient tachypnea of the newborn, aquaporin-1, aquaporin-5, aquaporins

## Abstract

Transient tachypnea of the newborn (TTN) is a common cause of neonatal respiratory distress, yet the role of aquaporins in its pathophysiology remains unclear. We aimed to evaluate serum aquaporin-1 and aquaporin-5 levels in neonates with TTN and their association with clinical characteristics and respiratory support parameters. This prospective study included 49 neonates (27 controls, 22 with TTN). Serum aquaporin-1 and aquaporin-5 levels were measured on the first day of life (T0) in both groups and repeated on day 3 (T3) in the TTN group using an enzyme-linked immunosorbent assay. Clinical data and respiratory support requirements were recorded. Group comparisons, correlation analyses, and multivariable regression were performed. Baseline aquaporin-1 levels were lower in the TTN group than in controls (*p* = 0.024) and decreased further at T3 (*p* = 0.004). Aquaporin-5 levels were similar at T0 and increased non-significantly at T3. Aquaporin-1 decline was greater in infants requiring prolonged oxygen therapy, noninvasive mechanical ventilation, or higher mean airway pressure, and was inversely correlated with ventilation duration and mean airway pressure at day 3. Aquaporin-5 levels were higher in infants requiring shorter respiratory support. Serum aquaporin-1 and aquaporin-5 levels show time-dependent changes during the early postnatal period and may be associated with clinical course and respiratory support requirements in TTN.

## 1. Introduction

Transient tachypnea of the newborn (TTN) is the most common cause (0.5–4%) of respiratory distress in term and late preterm infants [1]. Although TTN is usually benign and resolves within 3–4 days, delayed resolution may result in significant morbidity. Severe or prolonged TTN may require respiratory support, intravenous fluids, antibiotics, feeding interruption, and maternal–infant separation [2]. As no specific test exists to confirm or predict TTN severity, the diagnosis is based on exclusion. Risk factors for TTN include cesarean delivery, male sex, maternal history of asthma and diabetes, low gestational age, and macrosomia [2,3,4]. TTN occurs due to delayed postnatal reabsorption of lung fluid secreted by alveolar cells during fetal life [2,3,5]. During fetal life, lung fluid secretion is driven predominantly by active chloride transport, whereas at birth the pulmonary epithelium switches to a sodium-absorptive phenotype. The activation of epithelial sodium channels (ENaC) and basolateral Na^+^/K^+^-ATPase generates an osmotic gradient that promotes water reabsorption from the alveolar space. Impairment of this transition delays lung fluid clearance, disrupts gas exchange, and contributes to the development of TTN [3,5,6].

Aquaporins (AQPs) are integral membrane proteins that regulate cellular fluid balance. These approximately 30 kDa proteins facilitate transmembrane diffusion of water and small solutes by increasing membrane permeability [7]. Thirteen AQPs have been identified and classified into classical aquaporins (0, 1, 2, 4, 5, 6, 8), aquaglyceroporins (3, 7, 9, 10), and superaquaporins (11, 12), the latter with incompletely defined functions. AQPs are expressed in various tissues and organs [7,8]. Previous studies have implicated AQP2 in nephrogenic diabetes insipidus, AQP0 in cataracts, and anti-AQP4 antibodies in neuromyelitis optica [8,9].

Four types of AQPs have been identified in the lungs. AQP1 is expressed in the vascular endothelium and pleura, AQP3 in the basal cells of large airways, AQP4 in the upper airway and bronchial epithelium, and AQP5 in type I alveolar epithelium and submucosal glands [10,11]. Among these, AQP1 and AQP5 are thought to mediate water transport across epithelial and endothelial barriers in the peripheral lung [12,13]. Altered AQP1 and AQP5 expression has been associated with pulmonary fluid absorption and lung pathology [10,11,14]. Although increased AQP5 expression has been reported in TTN, the role of AQP1 remains unclear. In addition, serum levels of AQP1 and AQP5 have not been clearly defined. The aim of this study was to evaluate serum AQP1 and AQP5 levels in neonates with TTN and their association with clinical characteristics and respiratory support parameters.

## 2. Results

A total of 49 infants were included in the study, with 27 in the control group and 22 in the TTN group. There were no statistically significant differences between the groups in sex, mode of delivery, birth anthropometric measurements, or 1-min Apgar score. However, the proportions of male infants and cesarean deliveries were higher in the TTN group. Gestational age was significantly lower in the TTN group (*p* = 0.010). Maternal age, hemoglobin level, body mass index (BMI), and gestational weight gain were similar between the groups. The characteristics of the study groups are shown in Table 1.

**Figure 1:**
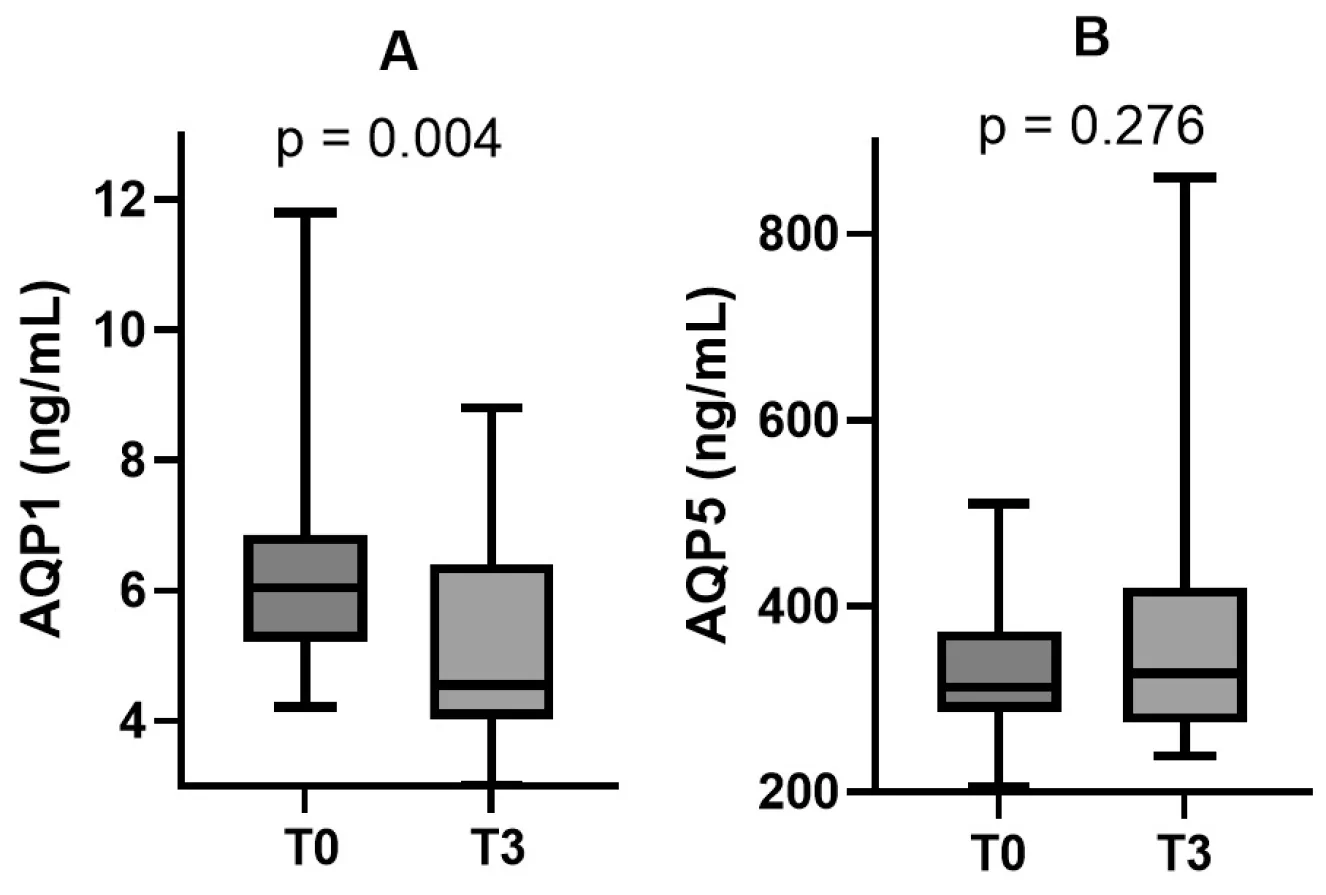
Serum AQP1 (**A**) and AQP5 (**B**) levels at T0 and T3 in infants with TTN.

Mean serum AQP levels were compared between the groups. Baseline (T0) AQP1 levels were higher in the control group (6.96 ± 1.1 ng/mL) than in the TTN group (6.46 ± 1.8 ng/mL) (*p* = 0.024). In the TTN group, T0 AQP1 levels were significantly higher than day 3 (T3) levels (5.11 ± 1.6 ng/mL) (*p* = 0.004). T0 AQP5 levels did not differ between the control (328.5 ± 36.6 ng/mL) and TTN (333.0 ± 78.8 ng/mL) groups. In the TTN group, AQP5 levels at T3 (367.5 ± 141.3 ng/mL) were higher than at T0, but the difference was not significant (Table 2). The temporal changes in AQP1 and AQP5 levels in the TTN group are presented in Figure 1.

At T0 in the control group, a positive but not significant correlation was observed between AQP1 and AQP5 levels (Pearson’s r = 0.347, *p* = 0.076). In the TTN group, moderate positive correlations were observed at T0 (Pearson’s r = 0.436, *p* = 0.043) and T3 (Spearman’s ρ = 0.625, *p* = 0.002).

In the control group, T0 AQP1 levels did not differ by sex (*p* = 0.277) or mode of delivery (*p* = 0.123). Similarly, AQP5 levels showed no differences with respect to sex (*p* = 0.915) or mode of delivery (*p* = 0.474) (Supplementary Table S1). However, in the TTN group, T3 AQP1 levels were lower than T0 in male infants and those delivered by cesarean section (*p* = 0.007 and *p* = 0.006, respectively; Table 3).

In the TTN group, the mean length of hospital stay was 5.3 ± 1.2 days (range 3–8). The mean duration of oxygen therapy was 42.2 ± 27 h (range 16–110). Non-invasive mechanical ventilation (NIMV) was administered to 20 infants, with a mean duration of 25.1 ± 19.4 h (range 5–80). The mean airway pressure (MAP) during NIMV was 7.1 ± 1.4 cm H_2_O (range 6–9). The mean fraction of inspired oxygen (FiO_2_) was 27.0 ± 6.4% (range 21–40). The mean duration of intravenous fluid therapy was 49.0 ± 17.3 h (range 24–72). The TTN group was further analyzed according to these variables. T3 AQP1 levels were lower than T0 in infants who received oxygen therapy ≥24 h, underwent NIMV ≥24 h, or had a MAP >6 cm H_2_O (*p* = 0.001, *p* = 0.003, and *p* = 0.007, respectively). In addition, AQP1 levels at T3 were found to be significantly lower in these infants compared with the T3 values of the other infants (*p* = 0.029, *p* = 0.034, and *p* = 0.027, respectively). T3 AQP5 levels were higher than T0 in infants who received oxygen therapy <24 h and NIMV <24 h (*p* = 0.019 and *p* = 0.009, respectively; Table 4).

In the TTN group, T3 AQP1 levels were negatively correlated with NIMV duration and MAP (Spearman’s ρ = −0.449, *p* = 0.047; ρ = −0.452, *p* = 0.046, respectively). In addition, T0 AQP1 and T3 AQP5 levels were negatively correlated with the 1-min Apgar score (ρ = −0.456, *p* = 0.033; ρ = −0.593, *p* = 0.003, respectively) (Supplementary Table S2). Similarly, in the control group, lower T0 AQP1 levels were associated with lower 1-min Apgar scores (ρ = −0.460, *p* = 0.016).

To evaluate the potential confounding effects of gestational age, sex, and mode of delivery, multivariable linear regression analyses were performed for baseline (T0) serum AQP1 and AQP5 levels in the overall study population (*n* = 49). Gestational age, TTN status, sex, and mode of delivery were not identified as independent predictors of baseline serum AQP1 or AQP5 levels (all *p* > 0.05) (Table 5).

## 3. Discussion

In this study, serum AQP1 and AQP5 levels showed time-dependent changes and were associated with clinical characteristics. In the TTN group, positive correlations between AQP1 and AQP5 levels were observed at both T0 and T3. AQP1 levels were lower at baseline than in controls and further decreased at T3; this decline was greater in infants requiring more intensive respiratory support. AQP5 levels increased at T3 and were higher in infants with a milder clinical course. In addition, AQP levels were negatively correlated with respiratory support parameters and Apgar scores. To our knowledge, this is among the first studies to evaluate dynamic serum AQP1 and AQP5 levels in TTN and to explore their association with respiratory support parameters. However, subgroup and correlation analyses were exploratory in nature and should therefore be interpreted with caution.

During fetal life, effective clearance of lung fluid at birth allows air entry into the lungs and initiation of gas exchange. This process depends on epithelial secretion and reabsorption of fluid. During fetal life, an osmotic gradient generated by active Cl^−^ secretion in the distal airway epithelium facilitates water movement into the alveolar lumen [1,2]. Toward birth, epithelial ion transport properties change, and fluid secretion decreases. At delivery, the lung epithelium shifts to a Na^+^-absorptive phenotype. Na^+^ enters epithelial cells through apical ENaC and is transferred to the interstitium via basolateral Na^+^/K^+^-ATPase; the resulting osmotic gradient promotes water reabsorption. Impairment of this mechanism may prevent lung fluid clearance, disrupt alveolar gas exchange, and contribute to conditions such as TTN [2,3,5,6].

AQPs are expressed in many tissues and facilitate transcellular water transport in response to osmotic gradients. Previous studies have shown that AQP1, located in the apical and basolateral membranes of the microvascular endothelium, and AQP5, located in the apical membrane of type I alveolar epithelium, are the principal aquaporins responsible for water transport in the lung [10]. Bai et al. reported that osmotic water transport between the alveolar air space and capillaries was reduced tenfold in AQP1-null rats [12]. Ma et al. reported that osmotic water transport between the alveolar air space and capillaries was reduced by 30% in heterozygous rats, tenfold in homozygous AQP5-deficient rats, and approximately thirtyfold when both AQP1 and AQP5 were deficient [13]. King et al. demonstrated that airway wall thickness increased by 44% in control rats after saline infusion, whereas no change occurred in AQP1-null rats [15]. These studies indicate that AQP1 and AQP5 play an important role in osmotic water transport in the lung.

However, studies in rats by Ma et al. and Song et al. showed that alveolar fluid clearance in the early postnatal period was maintained despite the absence of AQP1 and AQP5 [13,16]. Active fluid clearance occurs through osmotically driven water movement. Water transport in the pulmonary epithelium is slower than in renal tissue because it occurs under near-isotonic conditions and at relatively low ion transport rates. In lung tissue, the relatively low demand for fluid movement and increased paracellular permeability may explain preserved alveolar fluid reabsorption despite reduced water permeability in AQP knockout models [17]. These findings suggest that aquaporins may function as secondary regulators that facilitate water movement in the presence of an osmotic gradient in TTN. However, these studies were conducted in different species, and the role of aquaporins in alveolar fluid reabsorption, their regulatory mechanisms, and related factors remain unclear.

The only study evaluating serum AQP1 levels was conducted by Hong et al. in adults with colon cancer, and the mean level in healthy controls was 4.2 ng/mL [18]. In our previous study in healthy term neonates, the mean cord blood AQP1 level was 11.1 ng/mL and was higher in neonates delivered by cesarean section [19]. In this study, AQP1 levels were lower than those reported previously. However, unlike the previous study, AQP levels were not measured at birth but after the 12th hour because of the TTN diagnostic process. Although no previous studies have evaluated serum AQP5 levels in neonates, a study of gastric aspirates from healthy term neonates reported that mode of delivery influenced AQP5 and β-ENaC gene expression and that AQP5 levels were higher after cesarean delivery [20]. In a study of neonatal tracheal aspirates, AQP5 expression was higher in infants with TTN than in those with RDS or abnormal chest radiographs [21]. Together, these findings suggest that changes affecting pulmonary adaptation may influence aquaporin regulation.

Studies in fetal rats have shown that pulmonary AQP1 expression increases during late gestation and is enhanced by maternal corticosteroids, whereas AQP5 expression increases after birth and is not affected by corticosteroids [22,23]. However, a study in sheep reported that maternal corticosteroids increased AQP5 expression [24]. These findings may reflect species-related differences in developmental regulation. In our study, relatively high AQP1 levels at birth decreased postnatally, whereas AQP5 levels increased during the early postnatal days. When considered together with developmental expression patterns, our findings may indicate that circulating AQP1 and AQP5 levels follow different temporal patterns during early postnatal adaptation. However, this interpretation should be made cautiously, as no previous studies have evaluated serum AQP5 levels. Furthermore, because follow-up serum AQP measurements were not performed in the control group due to ethical considerations, the extent to which normal postnatal physiological adaptation contributed to the temporal changes observed in AQP1 and AQP5 levels in the TTN group could not be determined.

In our study, no significant differences were found in the TTN group regarding baseline FiO_2_ levels or duration of intravenous fluid therapy. However, the decline in T3 AQP1 levels was greater in infants requiring more intensive respiratory support (≥24 h of oxygen therapy, ≥24 h of NIMV, and MAP >6 cmH_2_O). In addition, AQP5 levels increased in infants with a milder clinical course. In correlation analysis, T3 AQP1 levels were inversely related to NIMV duration and MAP. These observations may suggest a possible association between circulating AQP changes and ventilatory support requirements.

These changes may reflect underlying physiological adaptation processes. In addition, weak negative correlations between AQP levels and Apgar scores may indicate a possible association with impaired perinatal adaptation. However, these findings are based on unadjusted analyses and should be interpreted with caution. Experimental studies indicate that in models of high tidal volume ventilation, hyperoxia, and increased alveolar fluid load, AQP1 expression is downregulated in association with inflammation and pulmonary stress. Reduced expression of AQP1, AQP5, and Na^+^/K^+^-ATPase has also been reported to impair water transport capacity [14,25,26,27]. Overall, the alterations in AQP levels in TTN may reflect a regulatory response related to increased ventilatory support and pulmonary stress.

Aquaporins are increasingly recognized as dynamically regulated membrane proteins whose subcellular localization and membrane abundance are controlled by trafficking, internalization, and recycling mechanisms in response to physiological and pathological stimuli. However, whether AQPs are released into the circulation during pulmonary injury or stress, and whether circulating AQPs can subsequently reintegrate into cell membranes, remains unknown. Nevertheless, serum AQP levels may not directly reflect local pulmonary expression or alveolar fluid dynamics. Since lung tissue or bronchoalveolar samples were not available in our study, the relationship between circulating serum AQP levels and pulmonary membrane expression could not be directly evaluated. Therefore, our findings should be interpreted as circulating biological changes associated with pulmonary adaptation and clinical course during TTN rather than as direct indicators of local lung expression. Furthermore, because the intravenous fluid support, oxygen concentrations, and tidal volumes used in our patients were substantially lower than those employed in experimental models, the observed AQP alterations should be regarded as correlational rather than mechanistic findings.

AQP1 levels were lower in the TTN group than in the control group. At first glance, this finding could be attributed to the lower mean gestational age of the TTN group. However, in the multivariable linear regression analysis, gestational age, sex, and mode of delivery were not identified as independent determinants of serum AQP1 levels. In addition, in our previous study conducted in healthy term newborns, no significant association was found between serum AQP1 levels and gestational age or sex. Taken together, these findings suggest that the observed changes in AQP1 and AQP5 cannot be explained solely by demographic differences such as gestational age, sex, and mode of delivery. Nevertheless, because the TTN group had a lower gestational age than the control group, the possibility of residual confounding cannot be completely excluded.

Most infants in the TTN group were delivered by cesarean section. The lower catecholamine and cortisol response associated with cesarean delivery may influence AQP1 levels. In our previous study, we demonstrated that healthy term newborns delivered by cesarean section had higher AQP1 levels at birth. In contrast, in the present study, baseline AQP1 levels did not differ according to the mode of delivery, whereas the decline in AQP1 levels over time was more pronounced in male infants and those delivered by cesarean section. This finding may be related to disease severity. However, because male sex and cesarean delivery are well-established risk factors for TTN, these findings should not be interpreted independently of the clinical characteristics of the disease and should therefore be interpreted with caution.

This study has several limitations. First, it was conducted at a single center with a relatively small sample size, which may limit the generalizability of the findings and the statistical power of multivariable analyses. Second, serum AQP levels could not be directly correlated with pulmonary tissue expression or alveolar fluid dynamics because direct assessment of lung tissue is not feasible in neonates due to practical and ethical considerations. In addition, subgroup and correlation analyses were exploratory and should therefore be interpreted with caution. The absence of established reference ranges for serum AQP levels and the analytical variability associated with the ELISA method may also affect the interpretation of the results. Nevertheless, the dynamic assessment of AQP levels at two time points and the evaluation of their associations with clinical parameters represent strengths of the study.

In conclusion, serum AQP1 and AQP5 levels showed time-dependent changes during TTN and were associated with respiratory support parameters. These findings may reflect circulating biological changes associated with TTN and pulmonary adaptation rather than primary pathophysiological mechanisms. However, the relationship between circulating AQP levels and membrane-bound pulmonary AQP expression remains unclear. Further experimental and clinical studies are needed to clarify the relationship between circulating and membrane-bound AQPs and their biological significance, as well as to better define the roles of aquaporins in neonatal lung fluid regulation and TTN pathophysiology.

## 4. Materials and Methods

This prospective study was conducted in the neonatal unit of Kanuni Sultan Süleyman Training and Research Hospital in Istanbul, between May 2024 and October 2025. The study protocol was approved by the institutional ethics committee (KAEK/2023.11.161), and written informed consent was obtained from the parents of all newborns, in accordance with the Declaration of Helsinki.

Neonatal exclusion criteria were prematurity (<34 weeks), postmaturity (>42 weeks), 1- or 5-min Apgar score < 7, perinatal asphyxia, congenital or metabolic disease, cardiovascular disease, anemia, congenital infection, early-onset sepsis, pneumonia, meconium aspiration syndrome, and delayed transition. Maternal exclusion criteria included age <18 or >40 years, fever, severe anemia, obesity, malnutrition, chronic diseases, diabetes mellitus (DM), gestational diabetes mellitus (GDM), thyroid diseases, eclampsia, preeclampsia, hypertension, premature rupture of membranes, multiple pregnancy, and medication use during pregnancy.

The diagnosis of TTN was based on clinical and radiological findings after excluding other causes of respiratory distress (hypoglycemia, hypocalcemia, respiratory distress syndrome, polycythemia). Eligible infants developed respiratory symptoms within the first 6 h of life, persisting for at least 12 h (tachypnea >60 breaths/min, retractions, nasal flaring, grunting, cyanosis), and had radiological findings consistent with TTN (prominent central vascular markings, symmetrical perihilar congestion, interlobar fissure fluid, widening of the intercostal spaces, pulmonary hyperinflation, flattening of the diaphragmatic domes). A requirement of oxygen support for ≥6 h was also considered a diagnostic criterion.

Gestational age, gender, birth measurements (weight, length, head circumference), 1- and 5-min Apgar scores, mode of delivery, and maternal prenatal demographic characteristics were recorded. Respiratory rate, duration of tachypnea, ventilatory support, feeding status, length of hospitalization, laboratory findings, and treatment were recorded. Maternal data were obtained from medical records and physical examination. Gestational age was determined by ultrasonography and the date of the last menstrual period. In all TTN cases, blood samples were obtained for complete blood count, C-reactive protein (CRP), glucose, electrolytes, blood gas analysis, and cultures. Blood samples for AQP1 and AQP5 level measurements were collected on the first day of life in both the control and TTN groups; additionally, in the TTN group, sampling was repeated on the third day of life.

Blood gases, electrolytes, and glucose were measured using an ABL 800 Flex blood gas analyzer (Radiometer America Inc., Brea, CA, USA). Whole blood count was performed using the BC-6800 Hematological Analyzer (Mindray, Shenzhen, China). Biochemical parameters were analyzed using the cobas c 702 clinical chemistry analyzer (Roche Diagnostics, Mannheim, Germany).

All samples were centrifuged at 1600× *g* at 4 °C. Serum aliquots were separated for aquaporin analysis and stored at −80 °C. Serum AQP1 and AQP5 concentrations were measured in duplicate using enzyme-linked immunosorbent assay (ELISA) kits (AQP-1: Catalog No. 201-12-0625; AQP-5: Catalog No. 201-12-6672; Shanghai Sunred Biological Technology Co., Ltd., Shanghai, China) in accordance with the manufacturer’s instructions. Concentrations of AQP1 and AQP5 in the samples were calculated by interpolation from the corresponding standard curves.

### Statistical Analysis

Data were analyzed using SPSS Statistics software version 27. As no previous study has reported serum AQP levels in TTN, sample size estimation was based on published neonatal data on AQP5 expression, assuming a large standardized effect size. With a two-tailed α of 0.05 and a power of 0.80, a minimum of 21 neonates per group was required.

Descriptive variables were expressed as mean ± standard deviation, median, minimum, maximum, frequency, and percentage. The normality of data distribution was assessed using the Kolmogorov–Smirnov and Shapiro–Wilk tests. Student’s *t*-test was used for the comparison of normally distributed quantitative independent variables, while the Mann–Whitney U test was applied for non-normally distributed variables. Categorical variables were analyzed using the chi-square test, and Fisher’s exact test was used when chi-square assumptions were not met. Within-group comparisons were performed using a paired *t*-test or Wilcoxon signed-rank test, as appropriate. Correlations between quantitative variables were evaluated using Pearson or Spearman correlation analysis according to data distribution; Spearman’s correlation coefficient (ρ) was used for non-normally distributed variables (|ρ| < 0.30: weak; 0.30 ≤ |ρ| < 0.70: moderate; |ρ| ≥ 0.70: strong). Multivariable linear regression analysis was performed to identify independent predictors of AQP1 levels. A *p* value < 0.05 was considered statistically significant.

## Figures and Tables

**Table 1:** Demographics and clinical characteristics of study groups.

	Groups (*n*, %, Mean ± SD, Min-Max)			
	Control Group (*n* = 27)	TTN Group (*n* = 22)	Total (*n* = 49)	*p*
Gender Female Male	12 (24.5) 15 (30.6)	7 (14.3) 15 (30.6)	19 (38.8) 30 (61.2)	0.630 ^F^
Mode of Delivery VD CS	11 (22.4) 16 (32.7)	4 (8.2)) 18 (36.7)	15 (30.6) 34 (69.4)	0.746 ^F^
Gestation week	38.7 ± 1.2 (36.5–41)	37.7 ± 1.3 (35.4–41)	38.3 ± 1.3 (35.4–41)	0.010 ^T^
Birth weight (g)	3293 ± 444 (2500–4020)	3282 ± 479 (2465–4000)	3287 ± 459 (2465–4020)	0.931 ^T^
Birth length (cm)	49.9 ± 2.6 (43–54)	49.8 ± 1.8 (46–52)	49.8 ± 2.2 (43–54)	0.691 ^M^
Birth head circumference (cm)	35.1 ± 1.4 (32–37)	34.9 ± 1.3 (33–38)	35 ± 1.3 (32–38)	0.440 ^T^
Apgar score (1 min)	7.1 ± 0.6 (6–8)	7.2 ± 0.6 (6–8)	7.2 ± 0.7 (6–8)	0.624 ^M^
Maternal age (year)	27.7 ± 6.2 (19–40)	29.9 ± 6.4 (20–40)	28.7 ± 6.3 (19–40)	0.225 ^T^
Maternal Hb (g/dL)	10.8 ± 1.6 (8.1–14.6)	10.4 ± 1.4 (7.5–13.1)	10.6 ± 1.5 (7.5–14.6)	0.388 ^T^
Maternal BMI	24.2 ± 3.0 (19.8–29.8)	24.9 ± 2.6 (21.4–29.9)	24.5 ± 2.8 (19.8–29.9)	0.393 ^M^
Maternal weight gain (kg)	11.8 ± 3.6 (6.0–20.0)	11.7 ± 4.8 (5–24)	11.8 ± 4.1 (5–24)	0.924 ^T^

^F^: Fisher’s exact test; ^T^: Student’s *t*-test; ^M^: Mann-Whitney U test.

**Table 2:** Comparison of serum AQP1 and AQP5 levels between the control and TTN groups.

AQP1 (ng/mL)	Control Group (*n* = 27)	TTN Group (*n* = 22)	Total (*n* = 49)	
T0	6.96 ± 1.1 (4.83–9.99)	6.46 ± 1.8 (4.21–11.79)	6.73 ± 1.5 (4.21–11.79)	0.024 ^M^
T3		5.11 **±** 1.6 (2.96–8.80)		
*p*		0.004 ^W^		
AQP5 (ng/mL)				
T0	328.5 ± 36.6 (258.2–387.5)	333.0 ± 78.8 (205.2–510.0)	330.5 ± 58.8 (205.2–510.0)	0.791 ^T^
T3		367.5 ± 141.3 (238.9–860.3)		
*p*		0.276 ^W^		

^W^: Wilcoxon test; ^T^: Student’s *t*-test; ^M^: Mann-Whitney U test.

**Table 3:** Comparison of T0 and T3 AQP levels by gender and mode of delivery in the TTN group.

	TTN Group (*n*, Mean ± SD, Min-Max)					
AQP’s (ng/mL)	Gender			Mode of Delivery		
	Female (*n* = 7)	Male (*n* = 15)	*p*	VD (*n* = 4)	CS (*n* = 18)	*p*
AQP1						
T0	5.9 ± 1.2 (4.5–7.7)	6.7 ± 2.0 (4.2–11.8)	0.503 ^M^	5.7 ± 1.0 (4.2–6.7)	6.6 ± 1.9 (4.5–11.8)	0.580 ^M^
T3	4.5 ± 1.5 (3.0–6.9)	5.4 ± 1.7 (2.9–8.8)	0.298 ^M^	4.9 ± 0.6 (4.2–5.7)	5.1 ± 1.8 (2.9–8.8)	0.837 ^M^
*p*	0.156 ^W^	0.007 ^P^		0.125 ^W^	0.006 ^W^	
AQP5						
T0	310.3 ± 39.9 (258.6–369.1)	343.6 ± 90.9 (205.2–510.0)	0.503 ^M^	309.6 ± 77.1 (205.2–385.9)	338.2 ± 80.5 (226.1–510.0)	0.525 ^T^
T3	306.3 ± 25.0 (274.5–338.7)	396.1 ± 164.1 (238.9–860.3)	0.298 ^M^	348.0 ± 81.5 (240.4–413.8)	371.9 ± 152 (238.9–860.3)	0.967 ^M^
*p*	0.578 ^W^	0.135 ^W^		0.125 ^W^	0.580 ^W^	

^W^: Wilcoxon test; ^P^: paired *t*-test ^T^: Student’s *t*-test; ^M^: Mann-Whitney U test.

**Table 4:** Serum AQP1 and AQP5 levels at T0 and T3 according to clinical variables in the TTN group.

	AQP1 T0	AQP1 T3	*p*	AQP5 T0	AQP5 T3	*p*
O2 therapy duration <24 h (*n* =11) ≥24 h (*n* = 11)	6.4 ± 2.4 (4.2–11.7)	5.8 ± 1.9 (2.9–8.8)	0.373 ^P^	336.0 ± 89 (205–497)	413.9 ± 180 (238–860)	0.019 ^W^
	6.4 ± 2.4 (4.2–11.8)	4.3 ± 0.9 (3.2–6.3)	0.001 ^P^	330.0 ± 70 (249–510)	321.2 ± 68 (244–430)	0.770 ^P^
*p*	0.237 ^M^	0.029 ^T^		0.863 ^T^	0.217 ^M^	
NIMV duration <24 h (*n* = 12) ≥24 h (*n* = 8)	6.8 ± 2.3 (4.2–11.8)	5.8 ± 1.8 (3.0–8.8)	0.111 ^P^	329.9 ± 83 (205–497)	411.8 ± 173 (240–860)	0.009 ^W^
	6.2 ± 0.9 (4.8–7.7)	4.2 ± 0.6 (3.3–5.3)	0.003 ^P^	346.4 ± 75 (278–510)	323.4 ± 66 (244–430)	0.547 ^P^
*p*	0.545 ^T^	0.034 ^T^		0.659 ^T^	0.208 ^M^	
Mean airway pressure 6 cmH_2_O (*n* = 12) >6 cmH_2_O (*n* = 8)	6.9 ± 2.3 (4.2–11.7)	5.8 ± 1.7 (3.0–8.8)	0.081 ^P^	344.7 ± 80 (205–497)	415.2 ± 171 (240–860)	0.077 ^W^
	6.0 ± 0.8 (4.8–6.9)	4.2 ± 0.7 (3.2–5.3)	0.007 ^P^	324.3 ± 79 (249–510)	318.3 ± 67 (253–430)	0.858 ^P^
*p*	0.302 ^T^	0.027 ^T^		0.584 ^T^	0.157 ^M^	
FiO_2_ level <30% (*n* = 13) ≥30% (*n* = 9)	6.6 ± 2.1 (4.2–11.8)	5.3 ± 1.7 (3.0–8.8)	0.024 ^P^	349.8 ± 94 (205–510)	381.0 ± 174 (240–860)	0.635 ^W^
	6.2 ± 1.4 (4.7–8.9)	4.8 ± 1.4 (2.9–6.9)	0.074 ^P^	308.7 ± 42 (226–364)	348.0 ± 78 (238–446)	0.114 ^P^
*p*	0.920 ^M^	0.511 ^T^		0.238 ^T^	0.896 ^M^	
IV fluid duration <48 h (*n* = 16) ≥48 h (*n* = 6)	6.4 ± 2.1 (4.7–8.9)	5.4 ± 1.7 (2.9–8.8)	0.056 ^P^	327.5 ± 78 (205–497)	376.0 ± 162 (238–860)	0.159 ^W^
	6.5 ± 0.8 (5.3–7.7)	4.2 ± 0.7 (3.3–5.4)	0.010 ^P^	347.7 ± 84 (278–510)	344.8 ± 62 (273–430)	0.949 ^P^
*p*	0.920 ^M^	0.203 ^M^		0.825 ^M^	0.971 ^M^	

^W^: Wilcoxon test; ^P^: paired *t*-test; ^T^: Student’s *t*-test; ^M^: Mann-Whitney U test.

**Table 5:** Multivariable linear regression analyses of baseline serum AQP1 and AQP5 levels (*n* = 49).

	AQP1 B (95% CI)	*p*	AQP5 B (95% CI)	*p*
Gestational age	−0.033 (−0.394 to 0.328)	0.854	−4.447 (−19.267 to 10.373)	0.548
TTN status	−0.770 (−1.704 to 0.163)	0.103	−4.981 (−43.346 to 33.384)	0.795
Sex	0.652 (−0.230 to 1.534)	0.143	16.664 (−19.570 to 52.897)	0.359
Mode of delivery	0.720 (−0.260 to 1.700)	0.146	13.469 (−26.817 to 53.756)	0.504

Model fit (AQP1: F(4,44) = 1.517, *p* = 0.214, R^2^= 0.121, Adjusted R^2^ = 0.041; AQP5: F(4,44) = 0.473, *p* = 0.755, R^2^ = 0.041, Adjusted R^2^ = −0.046). AQP, aquaporin; CI, confidence interval; TTN, transient tachypnea of the newborn.

## Data Availability

The raw data supporting the conclusions of this article will be made available by the authors on request.

## Supplementary Materials

The following supporting information can be downloaded at: https://www.mdpi.com/article/10.3390/ijms27167243/s1.
