# Supplementary material for: Dynamic Changes in Serum Aquaporin-1 and Aquaporin-5 in Transient Tachypnea of the Newborn
Source: Int J Mol Sci. 2026 Aug 14;27(16):7243. doi: 10.3390/ijms27167243 (PMC13513632; doi:10.3390/ijms27167243)
Supplement: Supplementary file 1 [file ijms-27-07243-s001.zip › ijms-4456973-supplementary.pdf]

## Supplementary Tables

Supplementary Table S1. Baseline (T0) AQP1 and AQP5 levels by gender and mode of delivery in the control group

| Control Group (n, mean±SD, min-max,) | | | | | | |
|---|---|---|---|---|---|---|
| | Gender | | | Mode of delivery | | |
| | Female ($n$ = 12) | Male ($n$ = 15) | p | VD ($n$ = 11) | CS ($n$ = 16) | p |
| AQP1 T0 (ng/mL) | 6.7 ± 1.1 (4.8 - 9.3) | 7.2 ± 1.0 (6.1 - 9.9) | $0.277^M$ | 6.6 ± 0.8 (4.8 - 7.7) | 7.2 ± 1.2 (5.7 - 9.9) | $0.123^T$ |
| AQP5 T0 (ng/mL) | 327.6 ± 39.2 (263.0 - 386.3) | 329.1 ± 35.8 (258.2 - 387.5) | $0.915^T$ | 322.2 ± 38.3 (263.0 - 386.3) | 332.7 ± 36.0 (258.2 - 387.5) | $0.474^T$ |

$^M$: Mann–Whitney U test; $^T$: Student's t-test.

Supplementary Table S2. Correlations of clinical, laboratory, and maternal parameters with serum AQP in infants with TTN.

| | AQP1 | | | | AQP5 | | | |
|---|---|---|---|---|---|---|---|---|
| | T0 | | T3 | | T0 | | T3 | |
| | $\rho$ | p | $\rho$ | p | $\rho$ | p | $\rho$ | p |
| Gestational age (weeks) | 0.056 | 0.804 | 0.081 | 0.719 | -0.085 | 0.706 | -0.175 | 0.436 |
| Birth weight (g) | 0.232 | 0.300 | 0.080 | 0.725 | 0.242 | 0.278 | -0.130 | 0.563 |
| Apgar score (1min) | -0.456 | 0.033 | -0.207 | 0.356 | -0.023 | 0.919 | -0.593 | 0.003 |
| O2 therapy duration (h) | 0.282 | 0.203 | -0.232 | 0.298 | 0.264 | 0.234 | -0.009 | 0.968 |
| NIMV duration (h) | -0.115 | 0.629 | -0.449 | 0.047 | 0.089 | 0.708 | -0.339 | 0.143 |
| Mean airway press.($cmH_2O$) | -0.063 | 0.790 | -0.452 | 0.046 | -0.214 | 0.366 | -0.341 | 0.141 |
| $FiO^2$ level (%) | -0.115 | 0.610 | -0.302 | 0.172 | -0.235 | 0.292 | -0.112 | 0.621 |
| IV fluid duration (h) | 0.363 | 0.096 | -0.232 | 0.298 | 0.184 | 0.412 | 0.025 | 0.911 |
| pH | -0.261 | 0.241 | -0.032 | 0.888 | 0.207 | 0.355 | 0.077 | 0.733 |
| Hb | 0.008 | 0.970 | 0.195 | 0.385 | -0.091 | 0.686 | 0.111 | 0.622 |
| Na | 0.008 | 0.971 | 0.163 | 0.469 | -0.049 | 0.828 | 0.241 | 0.279 |
| Cl | 0.136 | 0.547 | -0.060 | 0.792 | 0.041 | 0.858 | 0.102 | 0.652 |
| Maternal Age | 0.098 | 0.664 | 0.017 | 0.940 | 0.136 | 0.545 | 0.005 | 0.982 |
| Maternal Hb | -0.064 | 0.777 | 0.079 | 0.727 | 0.154 | 0.495 | -0.040 | 0.861 |
| Maternal BMI | -0.193 | 0.390 | 0.098 | 0.663 | 0.135 | 0.549 | -0.252 | 0.258 |
| Maternal Weight gain | -0.079 | 0.728 | 0.247 | 0.267 | -0.029 | 0.897 | -0.034 | 0.880 |

NIMV: Non-invasive mechanical ventilation, $\rho$ = Spearman's rho.
